# Spectrophotometric method development and validation for simultaneous estimation of Anagliptin and Metformin HCl BY Q - Absorption ratio method in synthetic mixture

**DOI:** 10.1016/j.heliyon.2020.e03855

**Published:** 2020-05-06

**Authors:** Ruchi H. Majithia, Dr. Akruti Khodadiya, Vaibhav B. Patel

**Affiliations:** a SAL Institute of Pharmacy, Ahmedabad, Gujarat, 380060, India; b C. U. Shah College of Pharmacy and Research, India; c C.U. Shah University, Wadhwan City, Gujarat, 363030, India

**Keywords:** Anagliptin (ANA), Metformin HCl (MET), Q - Absorption ratio method, Iso - Absorptive point, Simultaneous estimation, Chemistry, Analytical chemistry, Spectroscopy, Inorganic chemistry, Organic chemistry, Pharmaceutical chemistry

## Abstract

A simple, accurate, precise and economical Q- Absorption Ratio spectrophotometric method was developed and validated for estimation of Anagliptin and Metformin HCl in synthetic mixture. Anagliptin and Metformin HCl showed an iso-absorptive point at 238 nm in distilled water. The second wavelength used was 233 nm which is λ_max_ of Metformin HCl in distilled water. The concentration of the drugs was determined by using ratio of absorbance at iso-absorptive point (λ_1_ = 238 nm) and at the λ_max_ of Metformin HCl (λ_2_ = 233 nm). This method is linear for both drugs; in range of 2–12 μg/mL at λ_1_ (R^2^ = 0.999) and at λ_2_ (R^2^ = 0.9998) for Anagliptin, and in the range of 5–30 μg/mL for Metformin HCl found at λ_1_ (R^2^ = 0.9995) and at λ_2_ (R^2^ = 0.9997). The % Recovery was 100.42–101.83 % of Anagliptin and 99.94–101.63 % of Metformin HCl by standard addition method. The LOD was found to be 0.201 μg/mL and 0.262 μg/mL for Anagliptin at λ_1_ and λ_2_ respectively. The LOD was found to be 0.320 μg/mL and 0.167 μg/mL for Metformin HCl at λ_1_ and λ_2_ respectively. The LOQ was found to be 0.610 μg/mL and 0.794 μg/mL for Anagliptin at λ_1_ and λ_2_ respectively. The LOQ was found to be 0.972 μg/mL and 0.506 μg/mL for Metformin HCl at λ_1_ and λ_2_ respectively. The method was found to be precise as % RSD was less than 2.00 in Repeatability, Interday and Intraday precision for Anagliptin and Metformin HCl. The % assay of analyte drugs in synthetic mixture was found to be 100.601% of Anagliptin and 100.206 % of Metformin HCl which showed good applicability of the developed method.

## 1 Introduction

**Figure 1:**
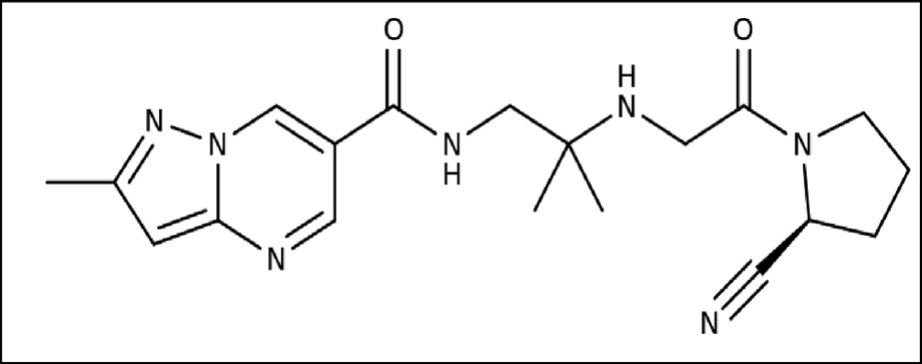
Chemical structure of Anagliptin.

Anagliptin, in form of Suiny® (100 mg tablets) is new drug formulation for type 2 diabetes therapy approved by the Japanese regulatory authority in 2014 [1]. Anagliptin, chemically N-[2-[[2-[(2S)-2-Cyanopyrrolodin-1-yl]-2-oxoethyl] amino]-2-methylpropyl]-2-methylpyrazolo [1, 5-a] pyrimidine-6-carboxamide (Figure 1) is a Dipeptidyl Peptidase 4 (DPP 4) inhibitor which is used in treatment of type 2 NIDDM [2]. Dipeptidyl Peptidase 4 enzyme breaks down the incretins GLP-1 gastrointestinal hormones released in response to a meal. By preventing GLP-1 inactivation, they are able to increase the secretion of insulin and suppress the release of glucagon by the alpha cells of the pancreas. This drives blood glucose levels towards normal level [3, 4]. This drug is not official in any of the pharmacopeia. Anagliptin is a very effective pill with minimum risk profile for type 2 diabetus mellitus and longer duration of action for treatment of type 2 non-insulin dependent diabetus mellitus disease [5]. A literature survey revealed that few methods are reported for determination of ANA, either alone or in combination [6], by spectrophotometric [7, 8, 9], HPLC [10], LC/MS [11].

**Figure 2:**
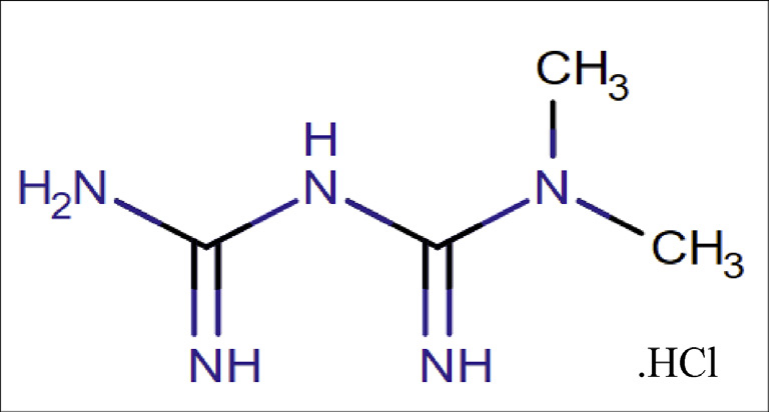
Chemical structure of Metformin HCl.

Metformin is chemically a 1-Caramimidamido-N, N-Dimethylmethanimidamide [12] (Figure 2) and has pharmacological action based on Biguanides category [13, 14]. It suppresses hepatic gluconeogenesis and glucose output from liver. This is the major action responsible for lowering blood glucose in diabetics. It is official in IP [15], BP [16], and USP [17]. Literature review revels that many spectrophotometric [18, 19], HPLC [20, 21], HPTLC [22] methods are reported for determination of Metformin hydrochloride (HCl), either alone or in combination ^[08]^.

The aim of the present work was to develop a Q- Absorption Ratio spectrophotometric method for simultaneous estimation of ANA and MET in combination. It is pertinent to note that, some of the published methods enabled estimation of drugs in combination products containing two drugs via zero and first order derivative spectrophotometric method and HPLC however, so far, not any Q- Absorption ratio spectrophotometric method was reported for the same. Hence, to achieve this aim an accurate Q- Absorption ratio method has been developed and successfully applied to synthetic mixture.

## 2 Materials and methods

### 2.1 Apparatus

Instrument used was of Shimandzu UV-1600 series with a pair of 1 cm matched quartz cells. Software used was UV Probe 4.2 series. A digital analytical balance (Wenstar DA14-222) and ultrasonic sonicator (Equitron) were used in the study. Validated pipette of 1, 2, 5 mL; volumetric flasks of 10,100 mL; beakers of 100, 250, 500 mL were made up of Borosil glass.

### 2.2 Chemicals and reagents

Drug sample of ANA and MET were provided as a gift sample by Intas Pharmaceutical Pvt. Ltd., Ahmedabad, India. Biciphage tablets (Metformin Hydrochloride 500mg) were purchased from local pharmacy store. Solvents like Distilled water were from E. Merck, Mumbai. All the chemicals reagents were of analytical Grade.

### 2.3 Preparation of standard stock solution

Accurately weighed quantity of 10 mg of ANA and 10 mg of MET were transferred into 100 mL volumetric flask individually. Initially about 50 mL distilled water was added to the flask respectively and sonicated. The volume was made up to the mark with distilled water to prepare stock solutions correspond to 100 μg/mL of ANA and 100 μg/mL of MET.

## 3 Methodology

**Figure 3:**
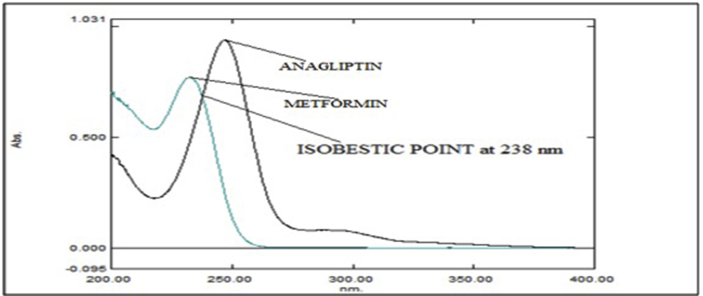
Overlay spectra of Anagliptin and Metformin HCl in Distilled water showing their iso-absorptive point at 238 nm.

The absorbance ratio method which obeys Beer's law at all wavelength, the ratio of absorbance at any single wavelengths is constant value independent of concentration or path length. At 238 nm, solutions of both drugs of same concentration exhibit identical absorbance and consequently with zero difference. Such wavelengths of equal absorptivity of the two species are called isobestic or iso-absorptive points [23].Q - Absorbance ratio method uses ratio of absorbance at two selected wavelengths, one which is an iso-absorptive point and other being the λ_max_ of one of two components. From overlay spectra of two drugs, it was evident that ANA and MET have an iso-absorptive point at 238 nm (λ_1_). The second wavelength used was 233 nm (λ_2_) of λ_max_ of MET. ANA and MET showed considerable absorbance at both wavelengths (Figure 3).

The concentration of two drugs of mixture in 1:5 ratio at 238 nm and 233 nm can be calculated using following equation [23]:

$${\text{C}}_{\text{x}}=\frac{{\text{Q}}_{\text{M}}-{\text{Q}}_{\text{y}}}{{\text{Q}}_{\text{x}}-{\text{Q}}_{\text{y}}}*\frac{{\text{A}}_{1}}{\text{ax}1}\;\;\;\;\;\;\;\;\;\;\;\;\;{\text{C}}_{\text{y}}=\frac{{\text{Q}}_{\text{M}}-{\text{Q}}_{\text{x}}}{{\text{Q}}_{\text{y}}-{\text{Q}}_{\text{x}}}*\frac{{\text{A}}_{2}}{\text{ay}1}$$

where, A_1_ and A_2_ are absorbance of mixture at 238 nm and 233 nm;

ax1 = A (Absorptivity, 1 %, 1 cm) of ANA at 238 nm (745.1)

ay1 = A (1 %, 1 cm) of MET at 238 nm (669.4)

ax2 = A (1 %, 1 cm) of ANA at 233 nm (549.8)

ay2 = A (1 %, 1 cm) of MET at 233 nm (770.8);

Cx and Cy are the unknown concentration of Anagliptin and Metformin HCl respectively in sample solution.

Q_M_ = A2/A1, Q_X_ = ax2/ ax1 and Q_Y_ = ay2/ay1

### 3.1 Preparation of test solution for assay

#### 3.1.1 Determination

Anagliptin/Metformin Hydrochloride is used in the ratio of 100/500mg for treatment of diabetes. Due to non-availability of product the condition of mixture was simulated by using Biciphage tablets (Metformin Hydrochloride 500mg) and API of ANA. Twenty tablets of Biciphage 500 mg tablets were weighed and triturated in a mortar pestle and powder equivalent to 500 mg of MET was taken into a 100 mL volumetric flask. To this flask, 100 mg of ANA API was added, to make concentration of ANA/MET in ratio of 1:5. The volume was adjusted to mark with distilled water to prepare test stock solutions correspond to 1000 μg/mL of ANA and 5000 μg/mL of MET, respectively. The contents of the flask were sonicated for 15 min to dissolve the active ingredients completely. The solution was filtered through a Whatman filter paper no. 41. From this 0.1 mL aliquot was transferred into a 10 mL volumetric flask and the volume was made up with distilled water. This test solution containing working concentrations of 2 μg/mL ANA and 10 μg/mL MET respectively, in mixture was analyzed for assay determination.

#### 3.1.2 Preparation of calibration curve

From working standard solution of ANA (100 μg/mL), aliquots of 0.2, 0.4, 0.6, 0.8, 1.0, 1.2 mL were transferred into series of 10 mL volumetric flask with the help of validated 1 mL pipette accurately and diluted up to mark with Distilled Water with the use of validated 10 mL pipette. This yielded solutions of 2, 4, 6, 8, 10 and 12 μg/mL of ANA. From working standard solution of MET (100 μg/mL), aliquots of 0.5, 1.0, 1.5, 2.0, 2.5 and 3.0 mL were transferred into series of 10 mL volumetric flask with the help of validated 1 mL pipette accurately and diluted up to mark with Distilled Water with the use of validated 10 mL pipette. This yielded solutions of 5, 10, 15, 20, 25 and 30 μg/mL of MET.

## 4 Method validation

The proposed method was validated as per ICH guidelines Q2 (R1) [24].

### 4.1 Linearity and range

Linearity was studied by preparing standard solutions at 6 different concentrations. The linearity range for ANA and MET were found to be 2–12 μg/mL and 5–30 μg/mL respectively. For each solution, the absorbance of ANA and MET were measured at λ_1_ and λ_2_. The calibration curves of absorbance versus concentration were plotted. The linearity of absorbance responses versus concentrations was demonstrated by linear regression analysis.

### 4.2 Precision

The precision of the proposed method was assessed as repeatability, intra-day precision and inter-day precision. Repeatability was performed by applying six replicates of sample solution. For intermediate precision, Intraday and Interday precision was performed by determining corresponding responses of six replicates on same and different days for test solution containing ANA (2 μg/mL) and MET (10 μg/mL). The results were reported in terms of % RSD.

### 4.3 Accuracy

Recovery studies were carried out by standard addition method. A known amount of standard ANA (1, 2 and 3 μg/mL) and MET (5, 10 and 15 μg/mL) similar to 50%, 100% and 150% of the label claim were added to test solution of ANA (2 μg/mL) and MET (10 μg/mL).

Same study was carried out three times, at each level of recovery.

### 4.4 LOD and LOQ

The LOD and LOQ of the developed method were calculated from the calibration curve using equation, LOD = 3.3∗σ/S and LOQ = 10∗σ/S. Where, σ = the standard deviation of y-intercepts of regression lines of six calibration curves, S = the average of the slopes of six calibration curves.

## 5 Result and discussion

### 5.1 Linearity

**Figure 4:**
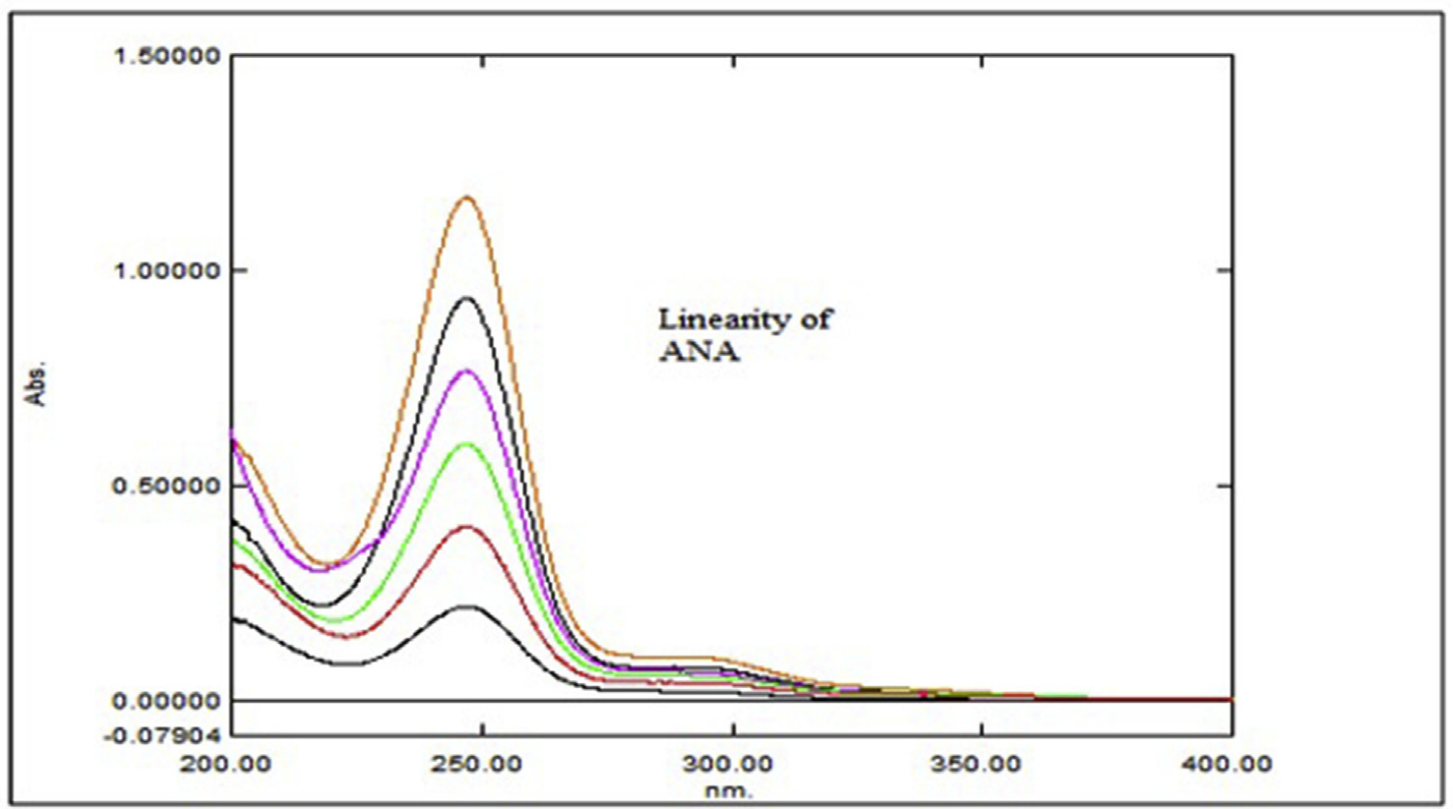
Overlain spectra for linearity of ANA (2–12 μg/mL).

**Figure 5:**
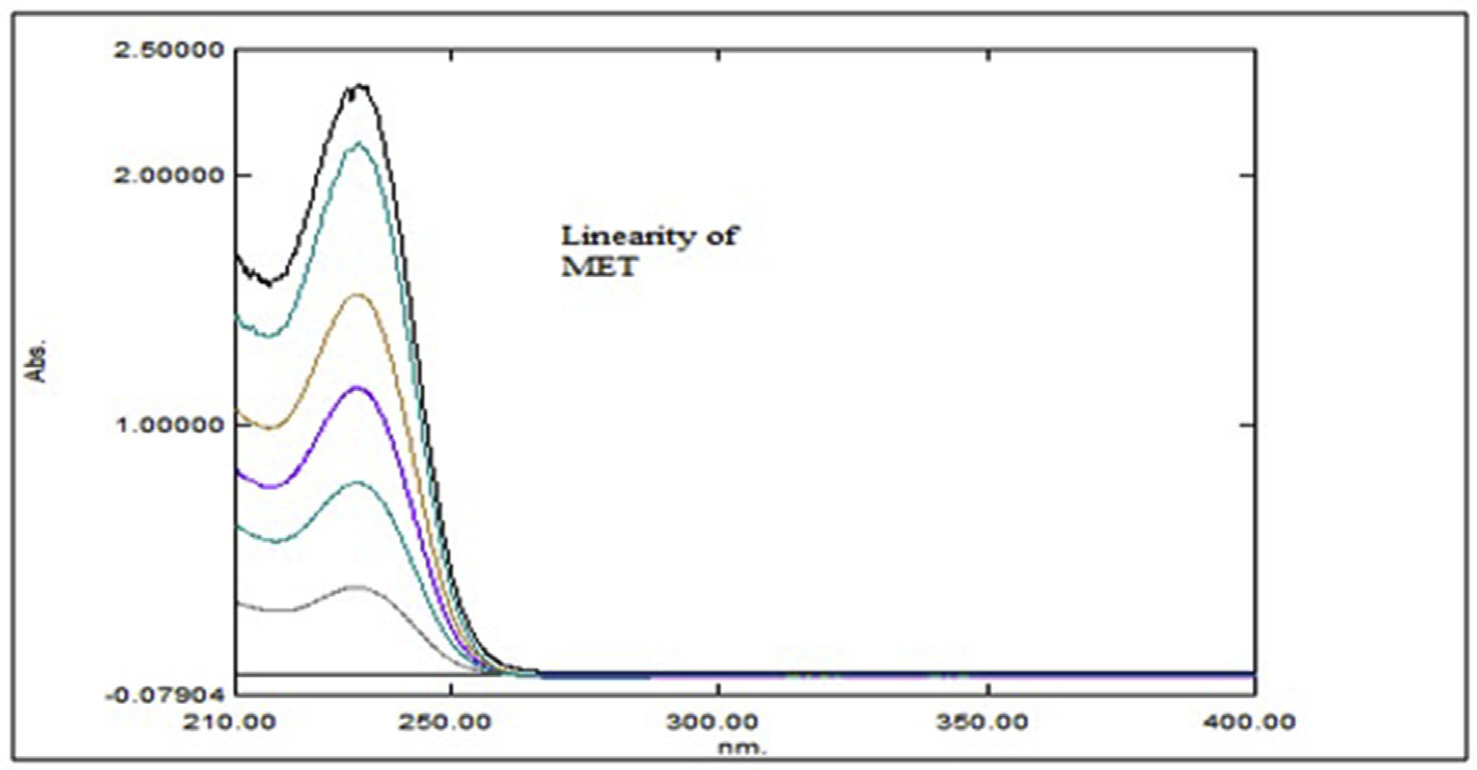
Overlain spectra for linearity of MET (5–30 μg/mL).

**Figure 6:**
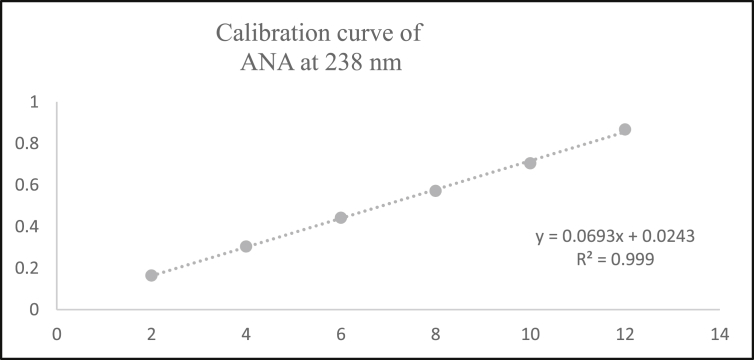
Calibration curve of ANA at λ_1_ = 238 nm.

**Figure 7:**
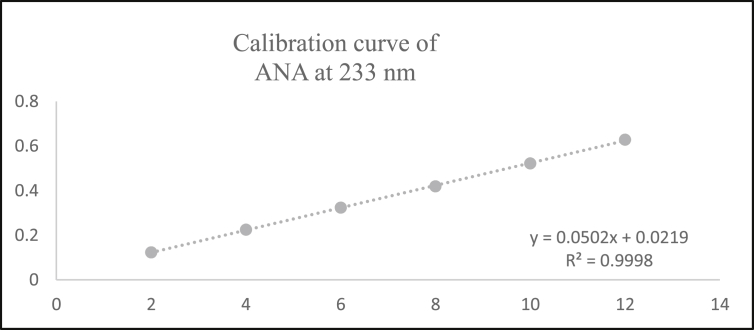
Calibration curve of ANA at λ_2_ = 233 nm.

**Figure 8:**
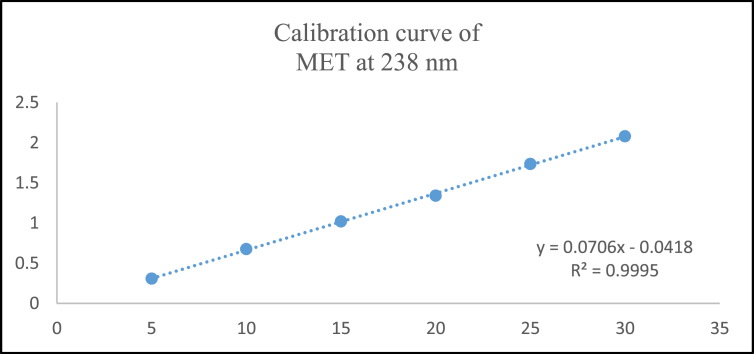
Calibration curve of MET at 238 λ_1_ = nm.

**Figure 9:**
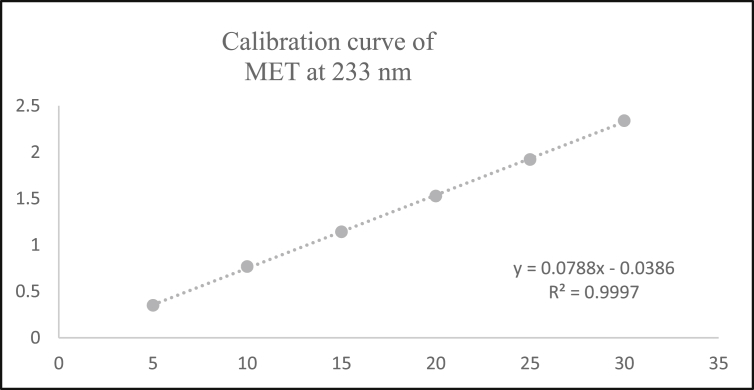
Calibration curve of MET at λ_2_ = 233 nm.

Aliquots of standard solution were applied in the concentration range 2–12 μg/mL and 5–30 μg/mL for ANA and MET respectively. The calibration curve obtained by the least square regression analysis between average absorbance and concentration showed linear relationship with a correlation coefficient R^2^ nearer to 0.999 for ANA and MET at λ_1_ and λ_2._ The linear regression equation obtained were y = 0.0693x + 0.0243 and y = 0.0502x + 0.0219 for ANA at λ_1_ and λ_2_ respectively. The linear regression equation obtained were y = 0.0706x - 0.0418 and y = 0.0788x - 0.0386 for MET at λ_1_ and λ_2_ respectively (Tables 1 and 2) (Figures 4, 5, 6, 7, 8, and 9).

∗Average of six determinations

**Table 1:** Linearity data of ANA and MET at λ_1_ as 238 nm.

Sr No.	ANA at λ_1_			MET at λ_1_		
	Conc (μg/mL)	Absorbance∗ ± SD	%RSD	Conc (μg/mL)	Absorbance∗ ± SD	%RSD
1	2	0.164 ± 0.002	1.552	5	0.308 ± 0.004	1.596
2	4	0.303 ± 0.004	1.546	10	0.675 ± 0.009	1.478
3	6	0.442 ± 0.007	1.670	15	1.020 ± 0.010	1.009
4	8	0.571 ± 0.004	0.770	20	1.340 ± 0.010	0.753
5	10	0.704 ± 0.005	0.757	15	1.735 ± 0.021	1.237
6	12	0.867 ± 0.012	1.437	30	2.078 ± 0.023	1.143

∗Average of six determinations (SD = Standard Deviation, % RSD = Percentage Relative Standard Deviation).

**Table 2:** Linearity data of ANA and MET at λ_2_ as 233 nm.

Sr No.	ANA at λ_2_			MET at λ_2_		
	Conc (μg/mL)	Absorbance∗ ± SD	%RSD	Conc (μg/mL)	Absorbance∗ ± SD	%RSD
1	2	0.122 ± 0.001	1.488	5	0.349 ± 0.001	0.463
2	4	0.224 ± 0.002	1.226	10	0.767 ± 0.004	0.579
3	6	0.323 ± 0.005	1.676	15	1.140 ± 0.014	1.284
4	8	0.419 ± 0.006	1.491	20	1.527 ± 0.008	0.540
5	10	0.522 ± 0.008	1.717	15	1.920 ± 0.013	0.710
6	12	0.628 ± 0.008	1.273	30	2.339 ± 0.027	1.182

∗Average of six determinations.

### 5.2 Precision

The % RSD of repeatability was found to be 1.147 and 1.161 of test solution containing ANA 2 μg/mL and MET 10 μg/mL. The % RSD of Intraday precision was found to be 1.241 and 1.192 at λ_1_ and λ_2_ respectively. The % RSD of Interday precision was found to be 1.403 and 1.367 at λ_1_ and λ_2_ respectively. Thus, confirming precision of the method (Table 3).

**Table 3:** Repeatability, intraday and interday precision of ANA and MET.

	Concentration of Test solution (μg/mL)		Absorbance∗ ± SD		%RSD	
	ANA	MET	At λ_1_ 238 nm	At λ_2_ 233 nm	At λ_1_ 238 nm	At λ_2_ 233 nm
Repeatability	2	10	0.831 ± 0.009	0.867 ± 0.009	1.161	1.147
Intraday Precision	2	10	0.890 ± 0.009	0.871 ± 0.010	1.192	1.241
Interday Precision	2	10	0.832 ± 0.011	0.880 ± 0.012	1.367	1.403

∗Average of six determinations.

### 5.3 Accuracy

Accuracy of the method was confirmed by recovery study from marketed formulation at three level of standard addition method. Percentage recovery for ANA was in range of 100.42–101.83 %, while for MET, it was found to be in range of 99.94–101.63 % (Table 4).

**Table 4:** Accuracy data of ANA and MET.

Drug	Amount of Test Solution (μg/mL)	Amount of Standard added (μg/mL)	Absorbance∗ ± SD	Total Amount Found (μg/mL)	Recovered amount (μg/mL)	% Recovery	% RSD
ANA	2	0	0.647 ± 0.007	2.008	---	100.42	1.081
	2	1	0.985 ± 0.011	3.054	1.054	101.83	1.195
	2	2	1.299 ± 0.016	4.029	2.029	100.74	1.260
	2	3	1.632 ± 0.027	5.062	3.063	101.26	1.673
MET	10	0	0.863 ± 0.009	10.008	---	100.08	1.089
	10	5	1.297 ± 0.015	15.032	5.032	100.21	1.204
	10	10	1.754 ± 0.018	20.327	10.327	101.63	1.041
	10	15	2.156 ± 0.023	24.986	14.986	99.94	1.071

∗Average of three determinations.

### 5.4 LOD & LOQ

The LOD was calculated by standard formula as given in ICH guidelines was found to be 0.201383 μg/mL and 0.26216 μg/mL for ANA at λ_1_ and λ_2_ respectively. The LOD was found to be 0.32089 μg/mL and 0.16716 μg/mL for MET at λ_1_ and λ_2_ respectively. The LOQ was calculated by standard formulae as given in ICH guidelines was found to be 0.61025 μg/mL and 0.79442 μg/mL for ANA at λ_1_ and λ_2_ respectively. The LOQ was found to be 0.97242 μg/mL and 0.50654 μg/mL for MET at λ_1_ and λ_2_ respectively.

### 5.5 Analysis of ANA and MET in test solution

**Figure 10:**
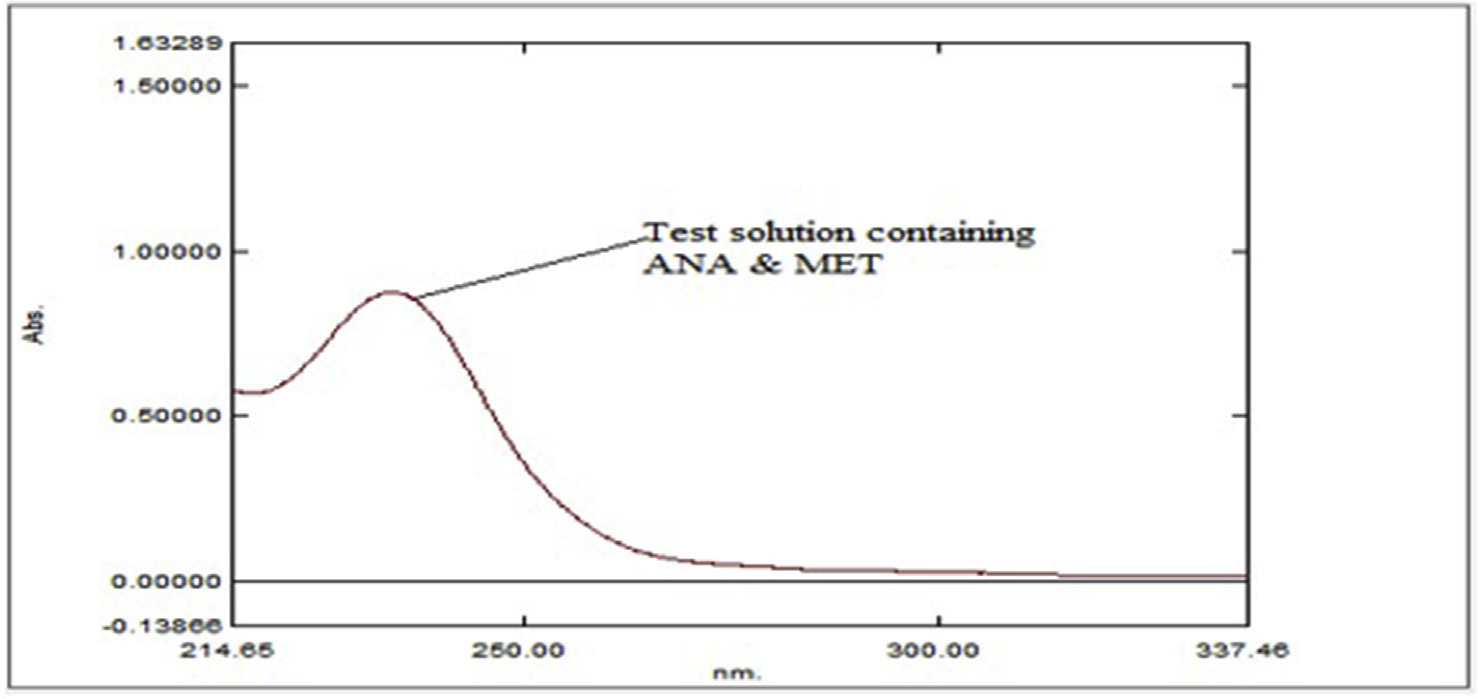
Spectra of Test solution of ANA (2 μg/mL) and MET (10 μg/mL).

The developed methods was applied to sample solution of synthetic mixture. The % Assay of ANA and MET was 100.601% and 100.206 % respectively of the labelled amount (Figure 10, Table 5).

**Table:** Summary of validation parameters.

Sr. No.	Parameter	ANA (238 nm)		MET (233 nm)	
		λ1	λ2	λ1	λ2
1	Specificity	Specific		Specific	
2	Linearity Range	2-12 μg/mL	5-30 μg/mL		
3	Regression Line equation	y = 0.0693x + 0.0243	y = 0.0502x + 0.0219	y = 0.0706x - 0.0418	y = 0.0788x - 0.0386
4	Correlation Coefficient	R2 = 0.999	R2 = 0.9998	R2 = 0.9995	R2 = 0.9997
5	Precision	% RSD			
	Repeatability	1.161		1.147	
	Intraday Precision	1.192		1.241	
	Interday Precision	1.367		1.403	
6	Accuracy (% Recovery)	100.42–101.83		99.94–101.63	
7	LOD (μg/mL)	0.201 μg/Ml	0.262 μg/mL	0.320 μg/mL	0.167 μg/mL
8	LOQ (μg/mL)	0.610 μg/mL	0.794 μg/mL	0.972 μg/mL	0.506 μg/mL

**Table 5:** Analysis of Tablet formulation.

Drug	Amount of drug Actual	Amount of drug Estimated	% Label claimed∗ ± SD	% RSD
ANA	2	2.012	100.601 ± 1.193	1.193
MET	10	10.02	100.206 ± 1.287	1.287

∗Average of six determinations.

## 6 Conclusion

The proposed spectrophotometric method is precise, specific, linear and accurate for the estimation of ANA and MET in synthetic mixture. The developed method is validated as per ICH guidelines Q2 R1. The method was successfully used for simultaneous estimation of both drugs in presence of each other.

## Declarations

### Author contribution statement

R.M. Hasmukhray:Conceived and designed the experiments; Performed the experiments; Analyzed and interpreted the data; Contributed reagents, materials, analysis tools or data; Wrote the paper.

A. Khodadiya and V.B. Patel: Analyzed and interpreted the data; Contributed reagents, materials, analysis tools or data; Wrote the paper.

### Funding statement

This research did not receive any specific grant from funding agencies in the public, commercial, or not-for-profit sectors.

### Competing interest statement

The authors declare no conflict of interest.

### Additional information

No additional information is available for this paper.
